# Integrated serum pharmacochemistry and investigation of the anti-gastric ulcer effect of Zuojin pill in rats induced by ethanol

**DOI:** 10.1080/13880209.2022.2098345

**Published:** 2022-08-07

**Authors:** Jiaying Zhang, Yi Yin, Qianqian Xu, Xiaoqing Che, Chen Yu, Yan Ren, Dongsheng Li, Juanjuan Zhao

**Affiliations:** a School of Pharmacy, Binzhou Medical University, Yantai, China; b College of Chemistry, Chemical Engineering and Biotechnology, Donghua University, Shanghai, China

**Keywords:** Multiple chemical constituents, antioxidation, anti-inflammation

## Abstract

**Context:**

Zuojin Pill (ZJP) has been used to treat gastrointestinal problems in China for hundreds of years.

**Objective:**

To discover more potential active ingredients and evaluate the gastroprotective mechanisms of ZJP.

**Materials and methods:**

An approach involving UPLC-Q-Orbitrap HRMS and serum pharmacochemistry was established to screen the multiple chemical constituents of ZJP. Male Sprague-Dawley (SD) rats were divided into six groups: normal control, ulcer control, omeprazole (30 mg/kg), and three ZJP groups (1.0, 2.0 and 4.0 g/kg). After oral treatment with ZJP or omeprazole for 7 days, all groups except the normal control group were orally administered 5 mL/kg ethanol to induce gastric ulceration. Histopathological assessment of gastric tissue was performed by haematoxylin and eosin staining. Antioxidant parameters and inflammatory mediators were determined using ELISA Kit and immunohistochemical analysis.

**Results:**

Ninety components were identified in ZJP. Among them, 23 prototypes were found in rat serum after oral administration of ZJP. The ulcer inhibition was over 90.0% for all the ZJP groups. Compared with the ulcer control rats, ZJP (4.0 g/kg) enhanced the antioxidant capacity of gastric tissue: superoxide dismutase (1.33-fold), catalase (2.61-fold), glutathione (2.14-fold), and reduced the malondialdehyde level (0.48-fold). Simultaneously, the ZJP meaningfully lowered the content of tumour necrosis factor-α (0.76-fold), interleukin-6 (0.66-fold), myeloperoxidase (0.21-fold), and nuclear factor kappa B (p65) (0.62-fold).

**Discussion and conclusions:**

This study showed ZJP could mitigate ethanol-induced rat gastric ulcers, which might benefit from the synergistic actions of multiple ingredients. The findings could support the quality control and clinical trials of ZJP.

## Introduction

Gastric ulcer is a common clinical gastrointestinal illness, with a lifetime prevalence of about 5–10% in the general population (Muazzam et al. 2021). The clinical manifestations include epigastric pain, abdominal distention, nausea, acid reflux and vomiting. Severe cases can cause perforation and gastrointestinal bleeding (Dovjak 2017). Pathogenic factors include poor diet, physical stress, tobacco abuse, prolonged administration of aspirin or other non-steroidal anti-inflammatory drugs (NSAIDs), excessive caffeine, and *Helicobacter pylori* infection (Satyanarayana 2006). In addition, gastric mucosal injury can be induced by excessive alcohol intake (Mousa et al. 2019). Currently, treatment options for gastric ulcers mainly include proton pump inhibitors, H2 receptor antagonists, antacids and triple therapy for the eradication of *Helicobacter pylori* (Kim et al. 2019). Nevertheless, some issues remain to be solved, such as long treatment cycles and severe side effects (Kangwan et al. 2019; Park et al. 2019). On the other hand, traditional Chinese medicine has been gradually recognised in gastric ulcer therapy because of its therapeutic effects and fewer side effects.

Zuojin Pill (ZJP), a classic drug pair firstly published in “Danxi prescription therapy” of the Yuan Dynasty, has been extensively applied in the clinical therapy of gastrointestinal ailments in China through the ages. ZJP is composed of the rhizome of *Coptis chinensis* Franch. (Ranunculaceae) (CC) and the near-mature fruit of *Tetradium ruticarpum* (A. Juss.) T. G. Hartley (Rutaceae) (TR) at a ratio of 6:1 (w/w) (Gao et al. 2010; Guo et al. 2019). CC has a bitter flavour and cold properties, whereas TR has a spicy flavour and warm properties. Thus, they complement each other and help jointly regulate the balance of deficiency (Shi et al. 2020; Wang et al. 2020). ZJP is recorded in the Chinese Pharmacopoeia (2020) and prescribed for purging fire, clearing liver heat, stopping vomiting, and relieving pain (Wang et al. 2020; Tong et al. 2021). Recent studies have shown that ZJP significantly affects gastric ulcers, ulcerative colitis, chronic atrophic gastritis, depression and hepatocellular carcinoma (Wang et al. 2015; Wei et al. 2021). However, the chemical and pharmacological foundations of ZJP for the treatment of gastric ulcers have not yet been clarified.

Due to the complexities of TCM components, illustrating its pharmacological material basis and mechanism of action is still challenging (Bai et al. 2018). However, recent studies have indicated that there are various active ingredients in CC and TR, such as berberine and quinolone alkaloids, which exhibit multiple pharmacological effects (Jiang et al. 2012; Liang et al. 2017). Nevertheless, few studies have investigated the potential active ingredients in ZJP that could be absorbed into the blood after oral administration.

The present study was undertaken to discover more constituents and potential active ingredients and estimate the gastroprotective effect of ZJP. First, the *in vitro* and *in vivo* constituents of ZJP were systemically identified based on a UPLC-Q-Orbitrap HRMS method and serum pharmacochemistry theory. Subsequently, the gastroprotective effects of ZJP were investigated in the rat model of ethanol-induced gastric injury. Meanwhile, pathological sections of gastric tissue from different groups were used to evaluate the gastroprotective activity of ZJP. Finally, the antioxidant parameters and inflammatory mediators were determined in gastric tissues to elucidate the potential mechanism of action of ZJP.

## Materials and methods

### Chemicals and materials

HPLC-grade acetonitrile and methanol were obtained from Merck KGaA (Darmstadt, Germany). HPLC-grade formic acid was supplied by Fisher Scientific (Waltham, MA, USA). Purified water was prepared in our laboratory using a Milli-Q system. CC and TR were obtained from the Tongrentang Drug Company (Yantai, China) and authenticated by Dr Shaoping Wang (School of Pharmacy, Binzhou Medical University, Yantai, China). Berberine hydrochloride, jatrorrhizine hydrochloride, evodiamine, rutaecarpine, evocarpine, dihydroevocarpine and limonin were obtained from Chengdu Must Bio-Technology Company, Ltd. (Sichuan, China). Magnoflorine and dehydroevodiamine were purchased from Yuanye Bio-Technology Company, Ltd. (Shanghai, China). The chemical purities of these compounds for reference were all over 98%.

### Animals

Male pathogen-free Sprague-Dawley rats (SPF, 220–250 g) were provided by Pengyue Co., Ltd. [Permit No. SCXK (Lu) 20180029, Jinan, China]. Rats were fed separately under a 12 h of light/dark cycle, at a temperature of 25 °C and 55% humidity with water and food available. All animal experiments were performed according to the guidelines for the Care and Use of Laboratory Animals of the National Institutes of Health (NIH), and the protocol was approved by the Animal Experiment Ethics Committee of Binzhou Medical University (No. BY2021-346).

### Preparation of ZJP

A mixture of CC 60 g and TR 10 g was extracted in 10-, 8- and 6-times volumes of 60% ethanol for 2 h each time, respectively. The resulting solution was collected and evaporated under reduced pressure to remove ethanol. Then the concentrated solution was lyophilised as powder, which was used as a ZJP for further *in vitro* and *in vivo* studies. Subsequently, the samples for CC or TR were also prepared using the same procedure.

### Sample preparation for identify chemicals *in vitro*

#### Preparation of standard solution

Berberine and other reference standards were precisely weighed to gain the stock solution of 0.1 mg/mL in methanol. Then a mixed standard solution of 20 ng/mL for each was prepared by diluting the corresponding stock solution using methanol. Stock solutions and mixed standard solutions were maintained at 4 °C before determination.

#### Preparation of sample solution

ZJP powder (0.1 g) was extracted using ultrasonication in 100 mL methanol-water (80–20, v/v) for 30 min, and the extracted solution was cooled and filtrated through a 0.22 µm microporous membrane. A solution of CC or TR was also prepared using the same procedure.

### Sample preparation for identify chemicals absorbed *in vivo*

Twelve rats were randomly assigned to two groups (*n* = 6): the blank group (5 mL/kg distilled water) and ZJP group (4.0 g/kg ZJP, determined as crude drug). ZJP and distilled water were orally administered once daily for 7 consecutive days. On day 7, the rats were fasted for 12 h and allowed free access to water before the last administration. Whole blood (500 µL) was collected from the orbital veins 0.5, 1, 2, and 3 h after gavage administration. The serum was prepared by centrifuging whole blood at 6000 rpm at 4 °C for 10 min after standing for 1 h and stored at −20 °C before analysis.

An aliquot of 75 µL serum samples at 0.5, 1, 2, and 3 h from the same rat was combined. The combined samples were vortexed for 1 min to mix well, and then 1.5 mL methanol was spiked and vortexed for another 5 min to obtain protein precipitation. The precipitated protein was separated by centrifuging at 13,000 rpm for 10 min at 4 °C. Then, the upper liquid was spiked into another centrifuge tube and dried under mild nitrogen flow at 35 °C. The remainder was dissolved in 150 μL of methanol-water (80–20, v/v). Then, the solution was rotated using a vortex metre for 5 min and centrifuged at 13,000 rpm for 10 min at 4 °C. Finally, 5 μL of the upper solution was used for the UPLC-HRMS analysis.

### UPLC-HRMS analysis

The components of the ZJP *in vitro* and *in vivo* were separated and identified using a Waters ACQUITY UPLC (Waters, Milford, USA) in tandem with a Q Exactive Orbitrap mass spectrometer (Thermo Scientific, Waltham, USA). The samples were analysed via an ACQUITY UPLC BEH Shield RP 18 column (2.1 × 100 mm, 1.7 μm) at 40 °C. The mobile phase was comprised of eluent A containing 0.1% formic acid/water and eluent B containing acetonitrile, at a 0.4 mL/min flow rate. The gradient separation procedure was set as follows: 0–15min, 7–25% B; 15–35 min, 25–70% B; 35–37 min, 70–90% B; 37–38 min, 90% B.

HRMS analysis was performed with an electrospray ion source with spray voltages at +3.8 kV and −2.8 kV for positive and negative modes, respectively. The temperatures of the capillary and auxiliary gas heater were 350 °C and 325 °C, respectively. The scan mode was full MS/data-dependent MS2 (ddMS2) with a scan range of *m/z* 100–1200, and the resolution of full MS was set at 70,000 FWHM, and 17,500 FWHM for ddMS2. 20, 40, and 60 eV were adopted for the stepped normalised collision energies. Software Xcalibur 4.1 was used for MS data acquisition and processing

### Experiment design against ethanol-induced gastric ulcer in rats

#### Animals grouping and gastric ulcer induction

In total, 36 male Sprague-Dawley rats were randomised into six groups (*n* = 6) as follows: normal control (distilled water), ulcer control (distilled water), omeprazole (30 mg/kg), low dose of ZJP (1.0 g/kg, determined as crude drugs), medium dose of ZJP (2.0 g/kg) and high dose of ZJP (4.0 g/kg). All rats in each group were administered the respective drug by gavage once a day for 7 consecutive days before ethanol induction. On the 7^th^ day, after 1 h of the last dose, all groups except the normal control group were orally administered a single dose of 5 mL/kg absolute ethanol to induce gastric ulceration.

After 1 h of ethanol administration, 10% chloral hydrate (3.5 mL/kg) was intraperitoneally injected into rats for anaesthetisation. The stomachs were then immediately taken out and sliced along the greater curvature. The contents of each stomach were rinsed mildly with cold saline. The liquid on the surface of the stomach was then dried with filter paper. The stomach was flattened with the mucosal surface facing upward, and digital photographs were taken of every stomach. After that, each stomach was divided into two parts: one was soaked in 10% formaldehyde for histopathological analysis and immunohistochemical study, and the other was kept at −80 °C for the determination of biochemical factors.

#### Morphological examination and assessment of gastroprotection

As mentioned previously, photographs of each stomach were taken by a digital camera to undergo gross evaluation for any signs of hyperaemia, haemorrhage, and ulcers in the stomach mucosa. The ulcer area was measured using ImageJ (National Institutes of Health, USA), and the relative ulcerated area (%) was counted. The ulcer inhibition percentage was calculated as follows: ulcer inhibition percentage = [the relative ulcerated area (ulcer control group) – the relative ulcerated area (each pre-treated group)]/the relative ulcerated area (ulcer control group) × 100%.

#### Histopathological examination

Stomach tissues were fixed in 10% formaldehyde and dehydrated by different concentrations of ethanol and xylene. The dehydrated stomach tissues were embedded in paraffin. Then the tissue blocks were sliced into 5 μm thick sections. The sections from different groups were stained with haematoxylin and eosin and inspected using a light microscope to observe any pathological changes at ×100 and ×400 magnification.

#### Measurement of oxidative stress markers in the gastric mucosa

The activity of superoxide dismutase (SOD) and catalase (CAT) and the content of malondialdehyde (MDA) and glutathione (GSH) in the gastric mucosa were tested using detection kits (Nanjing Jiancheng Bioengineering Institute, China).

#### Measurement of cytokines related with inflammatory in the gastric mucosa

Tumour necrosis factor-α (TNF-α) and interleukin-6 (IL-6) levels in the gastric mucosa were determined using ELISA kits supplied by MLbio (Shanghai MLbio Co., Ltd., China). The myeloperoxidase (MPO) concentration in the gastric tissue homogenate was evaluated by detection kits (Nanjing Jiancheng Bioengineering Institute, China).

#### Immunohistochemical analyses of NF-κB(p65)

The gastric sections were deparaffinized and rehydrated. Citrate buffer (10 mM, pH 6) was used for antigen retrieval at 95 °C for 20 min, and then 3% hydrogen peroxide was for consuming endogenous peroxidase at 37 °C for 30 min. The sections were then incubated with NF-κB (p65) polyclonal antibodies at a dilution ratio of 1:60 at 4 °C for 12 h. The corresponding secondary antibody was added to incubate after phosphate-buffered saline washing. The sections were then incubated with diaminobenzidine for 10 min, and counterstained with haematoxylin. Subsequently, the sections were dehydrated with a gradient of ethanol and fixed with neutral gum. Finally, the sections were examined under an optical microscope and the expression of NF-κB (p65) was assessed.

### Statistical analysis

All results were shown as the mean ± standard deviation. Data from different groups were analysed by SPSS Statistics version 26 (IBM, USA) using independent sample *t*-tests after analysis of variance. Statistical significance was set at *p* < 0.05.

## Results

### Characterisation of compounds in ZJP *in vitro*

**Figure 1.**
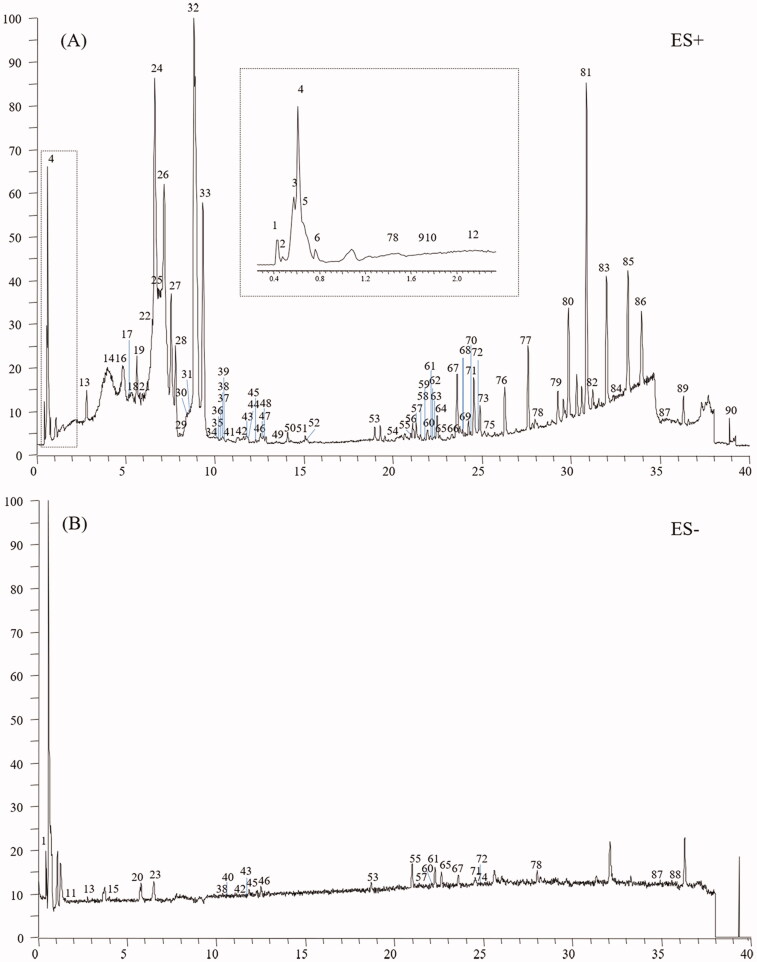
Total-ion chromatograms in the positive and negative modes of ZJP.

An in-house library, containing 205 compounds from the CC and TR, was established. Protoberberine alkaloids and aporphine alkaloids were discovered as the typical compounds for CC, whereas indole alkaloids, quinazoline alkaloids and limonoids were found for TR. Standard solution, containing jatrorrhizine (**28**), berberine (**32**), dehydroevodiamine (**25**), evodiamine (**67**), rutaecarpine (**71**), magnoflorine (**13**), evocarpine (**82**), dihydroevocarpine (**85**), and limonin (**54**), was analysed by the established UHPLC-Q-Orbitrap HRMS method to summarise the mass spectral fragmentation pathways and diagnostic ions. Subsequently, the sample solutions of ZJP, CC and TR were also analysed in both positive and negative ion modes. The total ion chromatograms of ZJP in the positive and negative modes are shown in Figure 1. Based on the proposed strategy, a total of 90 components, including 58 alkaloids, 11 flavonoids, 7 phenolic acids, 3 amino acids, 8 limonoids and 3 other types of compounds, were unambiguously identified or suspiciously characterised in ZJP. The identification information including retention time, formula, calculated mass, mass error and plant origin of the 90 compounds, are summarised in Table 1. Furthermore, the characterisation process of the five typical compounds is detailed below.

**Table 1.** Characterisation of the 90 compounds in ZJP by UHPLC-Q-Orbitrap HRMS.

Peak No.	t_R_ (min)	Formula	Ionization mode	Empirical mass (m/z)	Measured mass (m/z)	Error (ppm)	MS/MS fragments (m/z)	Compound identification	Source	Classification
1	0.47	C_6_H_14_N_4_O_2_	±	175.11852/ 173.10330	175.11937/ 173.10388	4.85/4.04	116.07, 70.07, 60.06/146.96, 131.08, 89.02	L-Arginine	CC, TR	A
2	0.52	C_9_H_11_NO_2_	+	166.08626	166.08658	1.93	149.06, 137.06, 121.06, 84.96	D-Phenylalanine	CC, TR	A
3	0.53	C_9_H_13_NO_2_	+	168.10191	168.10226	2.08	150.09, 146.03, 128.02, 119.05, 84.96	Synephrine	CC, TR	B6
4	0.6	C_10_H_13_N_5_O_4_	+	268.10403	268.10431	1.04	236.09, 214.08, 178.06, 169.05, 158.96, 135.08, 107.09, 90.98	Adenosine	CC, TR	B6
5	0.62	C_11_H_12_N_2_O_2_	+	205.09715	205.09741	1.27	188.07, 146.06, 118.07	L-Tryptophan	CC	A
6	0.68	C_19_H_18_NO_4_	+	324.12303	324.12314	0.34	308.09, 294.15, 280.10, 266.08	Demethyleneberberine	CC	B1
7	1.41	C_15_H_18_O_10_	+	359.09727	359.0979	1.75	163.04, 135.04, 89.04, 77.04	*trans*-caffeoylgluconic acid	CC	C
8	1.44	C_11_H_14_N_2_	+	175.12298	175.12323	1.43	144.08	*N*-Methyltryptamine	TR	B2
9	1.66	C_12_H_16_N_2_O	+	205.13354	205.13385	1.51	174.09, 162.09, 159.07	5-Methoxy-α-methyltryptamine	TR	B2
10	1.76	C_13_H_18_N_2_O	+	219.14919	219.14952	1.51	174.09, 143.07, 131.07	*N, N*-dimethyl-5-methoxytryptamine	TR	B2
11	1.94	C_7_H_6_O_4_	−	153.01824	153.01878	3.53	109.03	3, 4-Dihydroxybenzoic acid^Δ^	CC	C
12	2.15	C_13_H_16_N_2_O	+	217.13354	217.1338	1.19742	144.08	6-Methoxy-*N*-methyl-1, 2, 3, 4-tetrahydro-β-carboline	TR	B2
13	2.8	C_20_H_24_NO_4_	±	342.16998/ 340.15433	342.17026/ 340.15585	0.82/4.47	327.20, 297.11, 282.09, 265.09, 237.09/325.13, 310.11, 252.04, 224.05	Magnoflorine^Δ^	CC	B3
14	3.8	C_21_H_26_NO_4_	+	356.18563	356.18507	1.57	339.14, 288.92, 220.93, 206.12	Menisperine	CC, TR	B3
15	4.00	C_16_H_18_O_9_	−	353.08671	353.08826	4.39	191.06	Chlorogenic acid	CC, TR	C
16	4.88	C_26_H_34_O_12_	+	561.19425	561.19427	0.04	436.33, 288.93, 270.98, 220.94, 179.80, 135.00, 107.97, 90.95	Lanicepside A	CC	A
17	5.28	C_21_H_26_NO_5_	+	372.18055	372.18079	0.64	355.96, 342.87, 328.27	Stecepharine	CC, TR	B1
18	5.62	C_19_H_15_NO_4_	+	322.10738	322.10779	1.27	306.08, 294.10, 278.08	Berberrubine^Δ^	CC	B1
19	5.7	C_10_H_10_O_4_	+	195.06519	195.06541	1.13	177.05, 145.03, 131.97, 117.03, 113.96, 72.94	Methyl caffeate	TR	C
20	5.86	C_9_H_6_O_3_	−	161.02332	161.02376	2.73	133.03, 118.03, 85.03, 73.03, 61.99	7-Hydroxycoumarin	CC, TR	A
21	5.92	C_19_H_18_NO_4_	+	324.12303	324.12338	1.08	308.09, 294.08, 280.10, 266.08	2-Hydroxyjatrorrhizine	CC	B1
22	6.23	C_21_H_22_NO_5_	+	368.14925	368.14938	0.35	352.15, 336.12, 320.09	Stephabine	CC	B3
23	6.49	C_17_H_20_O_9_	−	367.10236	367.10373	3.73	193.05, 173.04, 134.04	5-*O*-Feruloylquinic acid^Δ^	CC	C
24	6.61	C_19_H_14_NO_4_	+	320.09173	320.09195	0.69	304.14, 290.08, 276.07, 262.09, 248.07	Coptisine^Δ^	CC	B1
25	6.65	C_19_H_15_N_3_O	+	302.12879	302.12894	0.50	286.10, 237.91, 164.92, 84.96	Dehydroevodiamine^Δ^	EF	B2
26	7.15	C_20_H_18_NO_4_	+	336.12303	336.1232	0.51	320.09, 306.08, 292.10	Epiberberine^Δ^	CC	B1
27	7.54	C_20_H_20_NO_4_	+	338.13868	338.13876	0.24	322.11, 308.09, 294.11, 280.10	Columbamine^Δ^	CC	B1
28	7.79	C_20_H_20_NO_4_	+	338.13868	338.13892	0.71	322.11, 308.09, 294.11, 280.10	Jatrorrihizine^Δ^	CC	B1
29	8.02	C_26_H_30_O_10_	+	503.19117	503.19189	1.43	137.06, 107.97, 90.98	Graucin A	TR	E
30	8.44	C_23_H_28_O_13_	+	512.15244	512.15582	6.60	336.12	Picroside II	CC	A
31	8.75	C_19_H_13_N_3_O	+	300.11314	300.11353	1.30	285.09, 255.91, 237.91	Evodianinine	TR	B2
32	8.83	C_20_H_18_NO_4_	+	336.12303	336.1232	0.51	320.09, 306.08, 292.10, 278.08	Berberine^Δ^	CC	B1
33	9.32	C_21_H_21_NO_4_	+	352.15433	352.15454	0.60	336.12, 322.11, 308.13, 294.11	Palmatine^Δ^	CC	B1
34	10.13	C_20_H_15_NO_4_	+	334.10738	334.10852	3.41	318.08, 304.06, 290.06	Worenine	CC	B1
35	10.16	C_21_H_20_NO_4_	+	350.13868	350.13895	0.77	334.11, 320.09, 306.11, 292.10	13-Methylberberine	CC	B1
36	10.18	C_19_H_21_NO_5_	+	344.14925	344.15024	2.88	302.13, 167.01	Salsoline A	TR	B6
37	10.22	C_28_H_34_O_15_	+	611.19705	611.19849	2.36	303.09, 153.02, 129.06, 107.97, 85.03, 71.05	Hesperidin	TR	D
38	10.41	C_27_H_30_O_16_	±	611.16066/ 609.14501	611.16144/ 609.14667	1.28/2.73	336.12, 208.04, 167.01/300.03, 271.02, 255.03, 243.03, 151.00, 61.99	Rutin	TR	D
39	10.53	C_22_H_24_NO_4_	+	366.16998	366.17102	2.84	350.14, 336.12, 322.14, 308.13	Dehydrocorydaline^Δ^	CC	B4
40	10.67	C_21_H_20_O_12_	−	463.0871	463.0889	3.89	300.03, 271.02, 255.03, 61.99	Hyperoside	TR	D
41	10.68	C_15_H_10_O_7_	+	303.04993	303.05066	2.41	286.10, 237.04, 245.05, 153.02, 107.97	Quercetin	CC, TR	D
42	11.29	C_29_H_34_O_17_	±	655.18688/ 653.17123	655.18744/ 653.17297	0.85/2.66	347.08/354.66, 329.03, 301.04, 287.02, 258.02	Syringetin-3-*O*-rutinoside	TR	D
43	11.84	C_28_H_32_O_15_	±	609.1814/ 607.16575	609.18231/ 607.16760	1.49/3.05	463.12, 301.07, 286.05/299.06, 284.03	Diosmin^Δ^	TR	D
44	11.91	C_23_H_24_O_12_	+	493.13405	493.13522	2.37	331.08, 315.05, 288.92, 270.05, 135.00, 90.98	Tricin-7-*O*-β-D-glucopyranoside	TR	D
45	12.29	C_28_H_32_O_16_	±	625.17631/ 623.16066	625.17743/ 623.16235	1.79/2.72	317.07/315.05, 271.02, 243.03, 146.97	Isorhamnetin-3-*O*-rutinoside	TR	D
46	12.55	C_22_H_22_O_12_	±	479.1184/ 477.10275	479.11932/ 477.10437	1.88/3.40	317.07/314.04, 271.03, 243.03	Isorhamnetin-3-*O*-b-D-galactoside	TR	D
47	12.57	C_30_H_33_N_3_O_11_	+	612.21879	612.2196	1.32	466.16, 304.11	Rutaecarpine-10-*O*-rutinoside	TR	B6
48	12.82	C_24_H_23_N_3_O_7_	+	466.16088	466.16098	0.21	304.11	Rutaecarpine-10-*O*-β-D-glucopyranoside	TR	B6
49	13.72	C_26_H_28_O_11_	+	517.17044	517.16998	−0.89	306.08, 278.08	Evodirutaenin	TR	E
50	13.95	C_18_H_19_NO_4_	+	314.13868	314.13943	2.39	177.05, 145.03, 121.06, 84.96	*N*-cis Ferulyltyramine	CC	B6
51	15.11	C_28_H_32_O_14_	+	593.18648	593.18738	1.52	509.89, 338.14, 260.09, 208.04, 167.01	Linarin	CC	D
52	15.16	C_14_H_13_NO_4_	+	260.09173	260.09222	1.88	245.07, 227.06, 216.07, 199.06, 184	Skimmianine^Δ^	TR	B4
53	18.71	C_26_H_30_O_9_	±	487.19626/ 485.18061	487.19684/ 485.18210	1.19/3.07	443.21, 396.60, 333.25, 286.22, 161.06, 107.97, 90.98/146.97, 123.04	12a-Hydroxylimonin	TR	E
54	20.11	C_26_H_30_O_8_	+	471.20134	471.20206	1.53	425.20, 319.06, 286.22, 161.06, 107.97	Limonin	TR	E
55	20.95	C_15_H_17_NO_3_	±	260.12812/ 258.11247	260.12845/ 258.11374	1.27/4.92	204.07/200.03, 188.03, 175.03	Ribalinine	TR	B5
56	20.99	C_19_H_15_N_3_O_2_	+	318.1237	318.12421	1.60	300.29, 278.90, 256.26, 107.97	14-Formyldihydrorutaecarpine^Δ^	TR	B2
57	21.4	C_26_H_28_O_9_	±	485.18089/ 483.16496	485.18137/ 483.16647	0.99/3.12	381.17, 353.18, 314.25, 421.16, 161.06, 135.04, 81.03	Evodol	TR	E
58	21.52	C_20_H_19_NO_6_	+	370.12851	370.12921	1.89	342.13, 327.11, 310.11, 278.08, 107.97	Chelerythrine	CC	B6
59	21.94	C_19_H_21_N_3_O	+	308.17574	308.17618	1.43	144.08, 134.06, 106.07	Evodiamide	TR	B2
60	22.1	C_26_H_28_O_8_	±	469.18569/ 467.17004	469.18671/ 467.17145	2.37/3.02	334.11, 167.01/423.18, 409.17, 312.70, 293.12, 251.00	Jangomolide	TR	E
61	22.26	C_18_H_15_N_3_O	±	290.12879/ 288.11314	290.12924/ 288.11450	1.55/4.72	273.10, 171.09, 144.08, 120.08/169.08, 145.04	Dehydrorutaecarpine	TR	B2
62	22.33	C_18_H_19_N_3_O	+	294.16009	294.16061	1.77	134.06, 90.98	*N*β-Femethylevodiamide	TR	B2
63	22.46	C_20_H_17_NO_5_	+	352.11795	352.11826	0.88	336.09, 322.07, 308.09, 294.08	8-oxo-epiberberine^Δ^	CC	B1
64	22.49	C_19_H_13_NO_5_	+	336.08665	336.08691	0.77	320.09, 306.08, 292.10	8-oxocoptisine^Δ^	CC	B1
65	22.63	C_28_H_32_O_10_	±	529.20682/ 527.19117	529.2077/ 527.19263	1.66/2.77	485.22, 469.19, 425.20, 367.16, 161.06, 105.07, 95.01, 485.18, 426.17, 383.15, 305.10	Rutaevineacetate	TR	E
66	23.52	C_15_H_16_O_4_	+	261.11214	261.11243	1.11	243.10, 215.11, 119.05, 107.97, 95.01, 90.98	Calodendrolide	TR	E
67	23.59	C_19_H_17_N_3_O	±	304.14444/ 302.12879	304.14447/ 302.13022	0.10/4.73	280.10, 191.85, 171.09, 161.07/169.08, 142.07	Evodiamine^Δ^	TR	B2
68	23.89	C_19_H_17_N_3_O_2_	+	320.13935	320.13986	1.59	177.07, 144.08	Goshuyuamide II	TR	B2
69	24.23	C_19_H_25_NO	+	284.20089	284.20117	0.99	186.09, 173.08	1-Methyl-2-[(*Z*)-4-nonenyl]-4(1H)-quinolone	TR	B5
70	24.27	C_19_H_19_N_3_O	+	306.16009	306.16046	1.21	134.06	Goshuyuamide I	TR	B2
71	24.53	C_18_H_13_N_3_O	±	288.11314/ 286.09749	288.11334/ 286.09857	0.69/3.77	273.09, 244.09, 185.07, 196.08/286.10, 218.87, 183.91, 116.93, 103.93, 92.93, 61.99	Rutaecarpine^Δ^	TR	B2
72	24.77	C_18_H_13_N_3_O_2_	±	304.10805/ 302.09240	304.10855/ 302.09378	1.64/4.57	289.08, 261.10, 220.93, 171.09, 134.06, 93.04/287.07, 276.08, 259.09, 169.08, 142.07	Hydroxyrutaecarpine	TR	B2
73	24.79	C_23_H_35_NO_2_	+	358.27406	358.27457	1.42	340.26, 186.09, 173.08	1-Methyl-2-[12-hydroxy-tridecyl]-4(1H)-quinolone	TR	B5
74	24.85	C_15_H_10_O_5_	−	269.04445	269.04559	4.24	180.39, 172.88, 165.02, 131.14, 116.93, 97.03, 79.02	Baicalein	CC	D
75	25.04	C_18_H_25_NO	+	272.20089	272.20081	−0.29	186.09, 173.08	1-Methyl-2-octyl-4(1H)-quinolone	TR	B5
76	26.26	C_19_H_27_NO	+	286.21654	286.21686	1.12	186.09, 173.08	1-methyl-2-nonyl-4(1H)-quinolone^Δ^	TR	B5
77	27.57	C_21_H_29_NO	+	312.23219	312.23236	0.54	186.09, 173.08	1-methyl-2-[(*Z*)-5-undecenyl]-4(1H)-quinolone	TR	B5
78	28.09	C_20_H_29_NO	±	300.23219/ 298.21654	300.23285/ 298.21747	2.20/3.12	173.08/189.04, 170.06, 158.06, 143.04, 61.99	1-Methyl-2-decyl-4(1H)-quinolone	TR	B5
79	29.24	C_23_H_31_NO	+	338.24784	338.24826	1.24	212.11, 186.09, 173.08	1-Methyl-2-[(4*Z*, 7*Z*)-4, 7-tridecadienyl]-4(1H)-quinolone	TR	B5
80	29.85	C_21_H_31_NO	+	314.24784	314.24805	0.67	200.11, 186.09, 173.08, 158.06, 132.06	1-Methyl-2- undecyl −4(1H)-quinolone^Δ^	TR	B5
81	30.84	C_23_H_33_NO	+	340.26349	340.26376	0.79	186.09, 173.08	l-Methyl-2-(8-tridecenyl)-4-(1H)-quinolone	CC	B5
82	31.19	C_23_H_33_NO	+	340.26349	340.26404	1.62	200.11, 186.09, 173.08	Evocarpine^Δ^	TR	B5
83	31.97	C_25_H_35_NO	+	366.27914	366.2796	1.26	186.09, 173.08	1-Methyl-2-[(6*Z*, 9*Z*)-6, 9-pentadecadienyl]-4(1H)-quinolone	TR	B5
84	32.02	C_22_H_33_NO	+	328.26349	328.26459	3.35	186.09, 173.08	1-Methy-2-dodecyl-4(1H)-quinolone	TR	B5
85	33.17	C_23_H_35_NO	+	342.27914	342.2796	1.34	186.09, 173.08, 160.08	Dihydroevocarpine^Δ^	CC, TR	B5
86	33.94	C_25_H_37_NO	+	368.29479	368.29532	1.44	186.09, 173.08	1-Methyl-2-[(*Z*)-9-pentadecenyl]-4(1H)-quinolone	TR	B5
87	35.22	C_24_H_37_NO	±	356.29479/ 354.27914	356.29544/ 354.28079	1.82/4.66	186.09, 173.08/348.56, 311.94, 189.04, 170.06, 157.05, 116.93, 81.56, 61.99	1-Methyl-2-tetradecyl-4-(1H)-quinolone	TR	B5
88	35.99	C_18_H_32_O_2_	−	279.23186	279.23312	4.51	234.31, 209.65, 205.16, 191.35, 180.88, 134.86, 114.90	Linoleic acid	CC	C
89	36.28	C_25_H_39_NO	+	370.31044	370.31107	1.70	186.09, 173.08	1-Methyl-2- pentadecyl −4(1H)-quinolone	TR	B5
90	38.91	C_10_H_10_O_4_	+	195.06519	195.06523	0.21	193.07, 180.04, 177.16, 162.03, 153.13, 135.12, 121.10, 107.09, 95.09, 81.07, 69.99	Ferulic acid	CC	C

A: Others (Amino acids/Lignans/Coumarins/Iridoids); B1: Protoberberine alkaloids; B2: Indole alkaloids; B3: Aporphine alkaloids; B4: Isoquinoline alkaloids; B5: Quinazoline alkaloids; B6: Other alkaloids; C: Phenolic acids; D: Flavonoids; E: Limonoids.

CC: The compound was from *Coptis chinensis* Franch.; TR: The compound was from *Tetradium ruticarpum* (A.Juss.) T.G.Hartley.

Δ: absorbed prototype components.

### Protoberberine alkaloids

**Figure 2.**
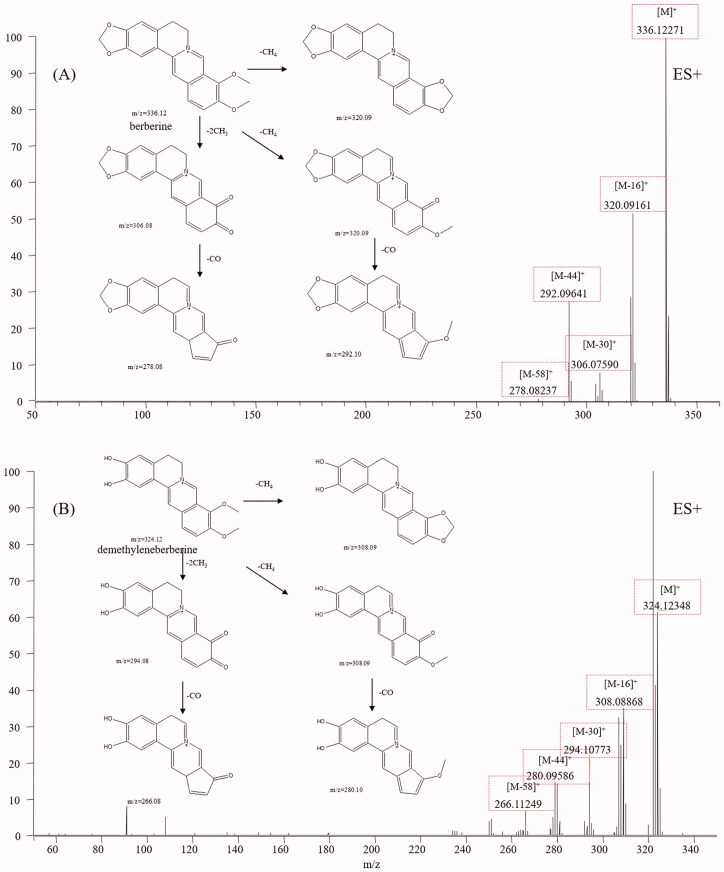
Typical mass spectrum and proposed fragmentation pathways of protoberberine alkaloids: berberine (A) and demethyleneberberine (B).

Protoberberine alkaloids are the main constituents of CC and have various bioactivities, including antibacterial, antioxidant, and antitumor (Li et al. 2020). Protoberberine alkaloids are composed of a tetracyclic ring structure owing to the dibenzo[*a,g*]quinolizidine structure (Leitao da-Cunha et al. 2005). The mass spectral fragmentation pathways of protoberberine alkaloids were studied by analysing jatrorrhizine (**28**) and berberine (**32**). The mass spectra and fragmentation pathways of berberine are presented in Figure 2A. Both berberine and jatrorrhizine displayed molecular ions M^+^ and their intense fragment ions, such as [M-16]^+^, [M-30]^+^, [M-44]^+^ and [M-58]^+^, indicating that these compounds exhibited common fragmentation pathways and diagnostic fragmentation ions. Considering the fragmentation pathways and accurate mass measurement within 5 ppm error, peaks **6, 17, 18, 21, 27, 34, 35, 39, 63** and **64** were assigned to demethyleneberberine, stecepharine, berberrubine, 2-hydroxyjatrorrhizine, columbamine, worenine, 13-methylberberine, dehydrocorydaline, 8-oxo-epiberberine, and 8-oxocoptisine, respectively. Typical mass spectra of demethyleneberberine and its fragmentation pathways are shown in Figure 2B, which is in accordance with our proposed fragmentation pathways.

### Aporphine alkaloids

**Figure 3.**
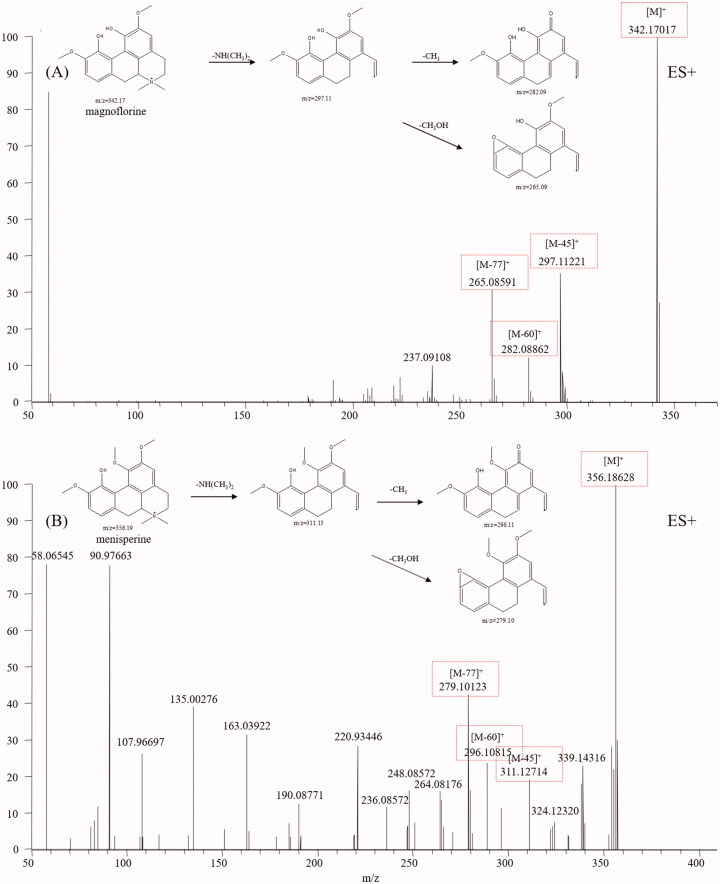
Typical mass spectrum and proposed fragmentation pathways of aporphine alkaloids: magnoflorine (A) and menisperine (B).

Aporphine alkaloids are another type of alkaloid from CC that display various biological activities, such as antitumor, antimicrobial, reversal of multidrug resistance, and antiviral activities (Liu et al. 2014). Aporphine alkaloids are characterised by a tetracyclic aromatic basic skeleton, including the phenol oxidative coupling of a benzylisoquinoline precursor (Ge and Wang 2018). Magnoflorine, a representative aporphine alkaloid found in CC, was first analysed. Molecular ions M^+^ and fragment ions, including [M-45]^+^, [M-60]^+^ and [M-77]^+^, were easily detected in the magnoflorine spectrum. The fragmentation pathways are shown in Figure 3A, from which we found aporphine alkaloids easily broke and recombined the side chain. According to the diagnostic fragmentation pathways [M-45]^+^, [M-60]^+^ and [M-77]^+^ and accurate mass measurement within 5 ppm error, peaks **14** and **22** were assigned as menisperine and stephabine, respectively. The typical mass spectra of menisperine and its fragmentation pathways are exhibited in Figure 3B.

### Indole alkaloids

**Figure 4.**
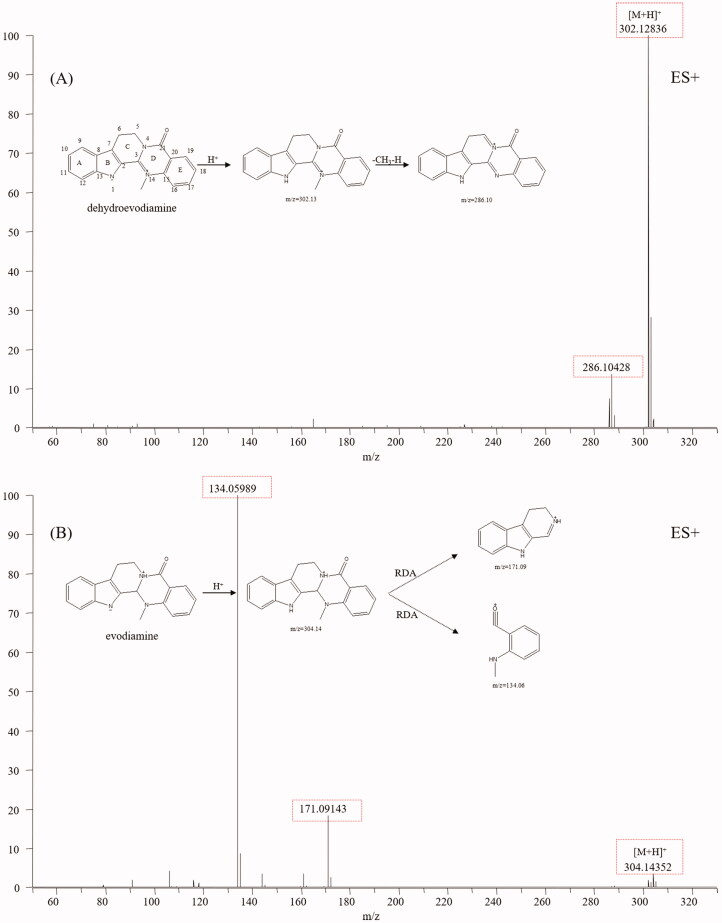
Typical mass spectrum and proposed fragmentation pathways of indole alkaloids: dehydroevodiamine (A) and evodiamine (B).

**Figure 5.**
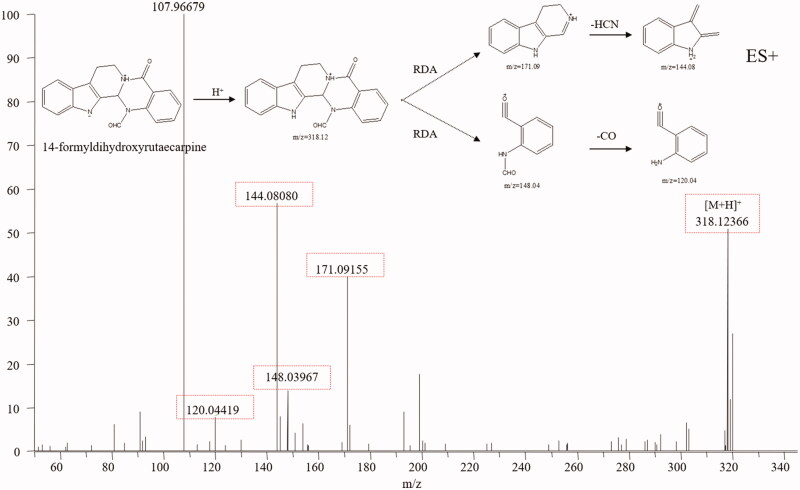
Typical mass spectrum and proposed fragmentation pathways of 14-formyldihydroxyrutaecarpine (B).

Indole alkaloids are the characteristic constituents of TR, including evodiamine and rutaecarpine, which show diverse bioactivities, such as anti-inflammatory, antimicrobial, anti-HIV, antioxidant, and anticancer activities (Tian et al. 2019). The typical structure of indole alkaloids contains a bicyclic structure formed by the fusion of a benzene ring and a five-membered pyrrole ring (Rosales et al. 2020). Dehydroevodiamine (**25**), evodiamine (**67**) and rutaecarpine (**71**) were used to summarise the mass spectral fragmentation pathways of the indole alkaloids. Dehydroevodiamine displayed diagnostic fragmentation ion m/z 286.10 (Figure 4A), which formed a big conjugation system by fusing with π-bonds and was rather stable, while both evodiamine and rutaecarpine displayed fragmentation ions *m/z* 171.09, 134.06 and 169.08, 120.04, which was caused by the split of ring-D (Figure 4B). The fragmentation pathways of indole alkaloids are summarised as follows (Kumar et al. 2016, 2018): (1) If a double bond existed between C-3 and C-14, no mass fragments of ring split were found because a stable conjugation structure was formed by π-bonds. If not, a fragment ion from reverse the Diels-Alder split of ring-D would be discovered. (2) If the bonds between C-2 and C-3, C-3, and C-14 break, ring-C and ring-D would not exist. No Diels-Alder cleavage is observed, and the broken active sites are amide and quaternary amide bond. (3) If only the ring-C is open, an amide bond, a quaternary amide bond, and a Diels-Alder cleavage will simultaneously appear. (4) For linear indoleamine alkaloids, the diagnostic ion m/z 160.1 (C_10_H_10_NO) could be obtained after the loss of the corresponding alkylamine group. Based on the diagnostic fragmentation ions and accurate mass measurements, peaks **8, 9, 10, 12, 31, 56, 59, 61, 62, 68, 70** and **72** were assigned to *N*-methyltryptamine, 5-methoxy-α-methyltryptamine, *N, N*-dimethyl-5-methoxytryptamine, 6-methoxy-N-methyl-1,2,3,4-tetrahydro-β-carboline, evodianinine, 14-formyldihydroxyrutaecarpine, evodiamide, dehydrorutaecarpine, *N*β-demethylevodiamide, goshuyuamide II, goshuyuamide I, and hydroxyrutaecarpine, respectively. Typical mass spectra and fragmentation pathways of 14-formyldihydroxyrutaecarpine are exhibited in Figure 5.

### Quinolone alkaloids

**Figure 6.**
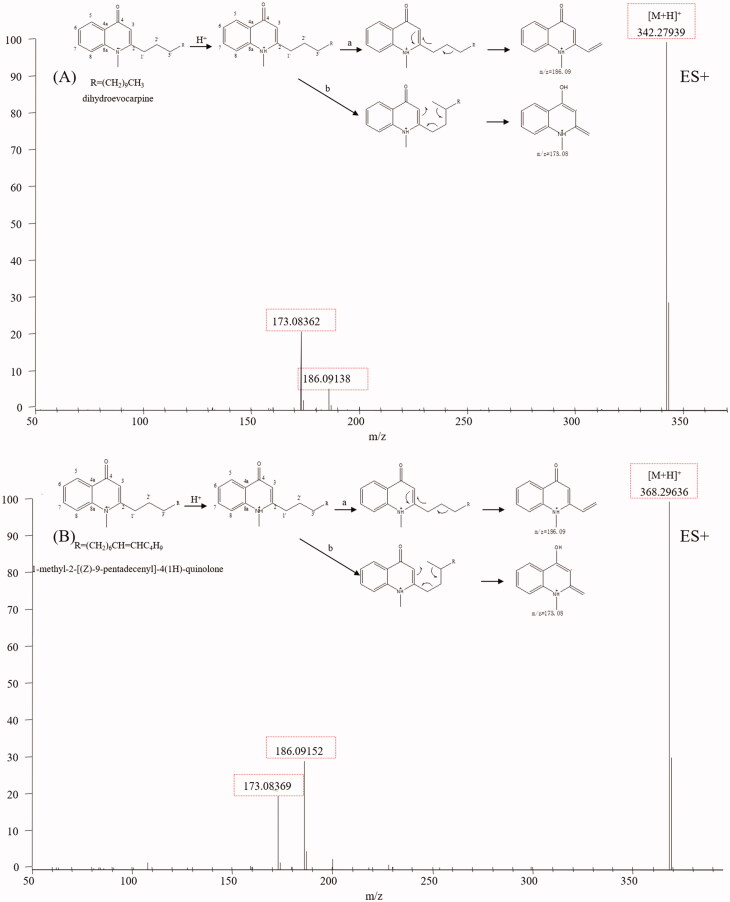
Typical mass spectrum and proposed fragmentation pathways of quinolone alkaloids: dihydroevocarpine (A) and 1-methyl-2-[(*Z*)-9-pentadecenyl]-4(1H)-quinolone (B).

Many quinolone alkaloids have been isolated from TR, some of which possess anti-*Helicobacter pylori*, anti-inflammatory and cytotoxic activities (Wang et al. 2013). The basic skeleton of quinolone alkaloids is identical except for the C-2 side chain (Huang et al. 2012). In this paper, evocarpine (**82**) and dihydroevocarpine (**85**) were used to study the fragmentation pathways of the quinolone alkaloids. Characteristic fragment ions including m/z 186.09 and *m/z* 173.08 were found in their mass spectra. Ions at *m/z* 186 arose from the electron rearrangement and chemical bond fracture between C-3, C-2, C-1′, C-2′ and C-3′, while ions at *m/z* 173 originated from the McLafferty rearrangement between γH at C-3′, C-3, C-2, C-1′, C-2′ and C-3′ (Figure 6A). Due to the diagnostic ions at *m/z* 186.09 and m/z 173.08, peaks **55, 69, 73, 75, 76, 77, 78, 79, 80, 81, 83, 84, 86, 87** and **89** were characterised as ribalinine, 1-methyl-2-[(*Z*)-4-nonenyl]-4(1H)-quinolone, 1-methyl-2-[12-hydroxy-tridecyl]-4(1H)-quinolone, 1-methyl-2-octyl-4(1H)-quinolone, 1-methyl-2-nonyl-4(1H)-quinolone, 1-methyl-2-[(*Z*)-5-undecenyl]-4(1H)-quinolone, 1-methyl-2-decyl-4(1H)-quinolone, 1-methyl-2-[(4*Z*,7*Z*)-4,7-tridecadienyl]-4(1H)-quinolone, 1-methyl-2-undecyl-4(1H)-quinolone, l-methyl-2-(8-tridecenyl)-4-(1H)-quinolone, 1-methyl-2-[(6*Z*,9*Z*)-6,9-pentadecadienyl]-4(1H)-quinolone, 1-methy-2-dodecyl-4(1H)-quinolone, 1-methyl-2-[(*Z*)-9-pentadecenyl]-4(1H)-quinolone, 1-methyl-2-tetradecyl-4-(1H)-quinolone and 1-methyl-2-pentadecyl-4(1H)-quinolone, respectively. Typical mass spectra of 1-methyl-2-[(*Z*)-9-pentadecenyl]-4(1H)-quinolone and its fragmentation pathways are exhibited in Figure 6B.

### Limonoids

**Figure 7.**
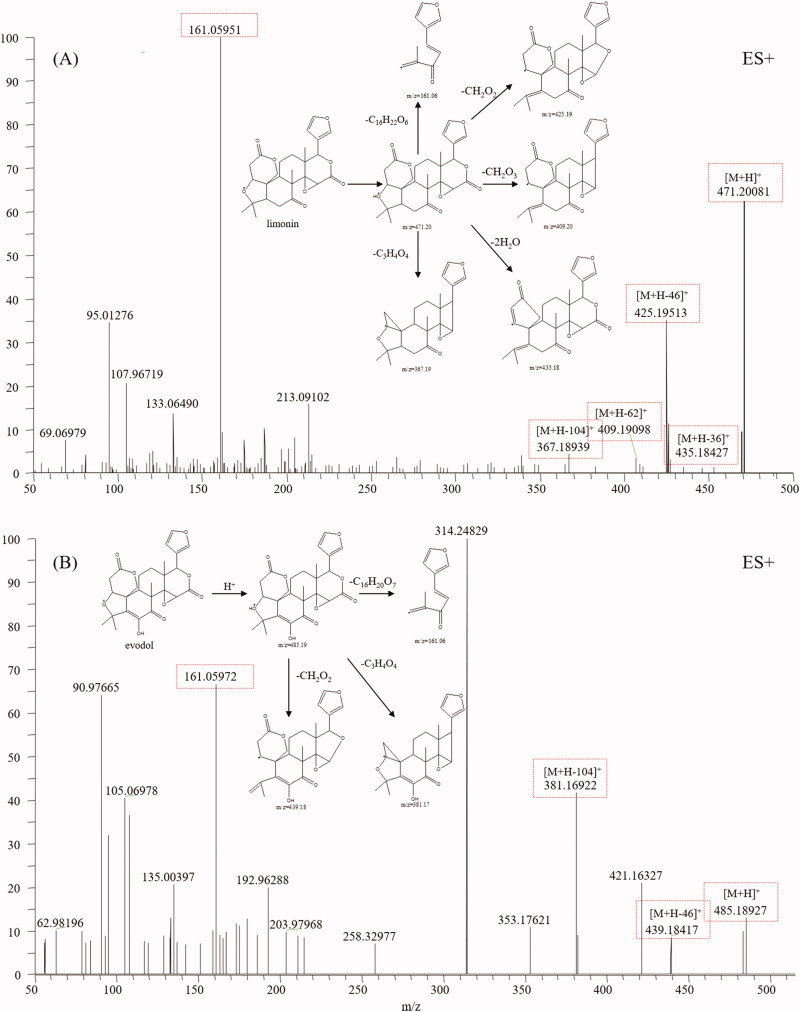
Typical mass spectrum and proposed fragmentation pathways of limonoids: limonin (A) and evodol (B).

Limonoids are highly oxygenated and polycyclic triterpenoids and are also the main constituents of TR (Shi et al. 2020). They have variable chemical structures and exhibit a wide spectrum of bioactivities, including anticancer, anti-inflammatory, neuroprotection, antibacterial, antioxidant activities (Fan et al. 2019). Limonin (**54**), a compound characterised by TR, was first analysed using HRMS to find the typical fragment ions of limonoids (Figure 7A). Limonin displayed quasi-molecular ions [M + H]^+^ and other intense fragment ions, such as, [M + H-H_2_O]^+^, [M + H-2H_2_O]^+^ and [M + H-CO_2_]^+^. Moreover, the fragment ion at *m/z* 161.0595 (C_10_H_9_O_2_) was deemed the diagnostic ion for limonoids according to our study and references. Based on the diagnostic fragmentation ions [M-17]^+^, [M-35]^+^, [M-43]^+^, [M-45]^+^, *m/z* 161.0595, and accurate mass measurements, peaks **29, 49, 53, 57, 60, 65,** and **66** were assigned to graucin A, evodirutaenin, 12α-hydroxylimonin, evodol, jangomolide, rutaevineacetate and calodendrolide, respectively. Typical mass spectra of evodol and its fragmentation pathways are presented in Figure 7B.

### Identification of the constituents absorbed in rat serum

**Figure 8.**
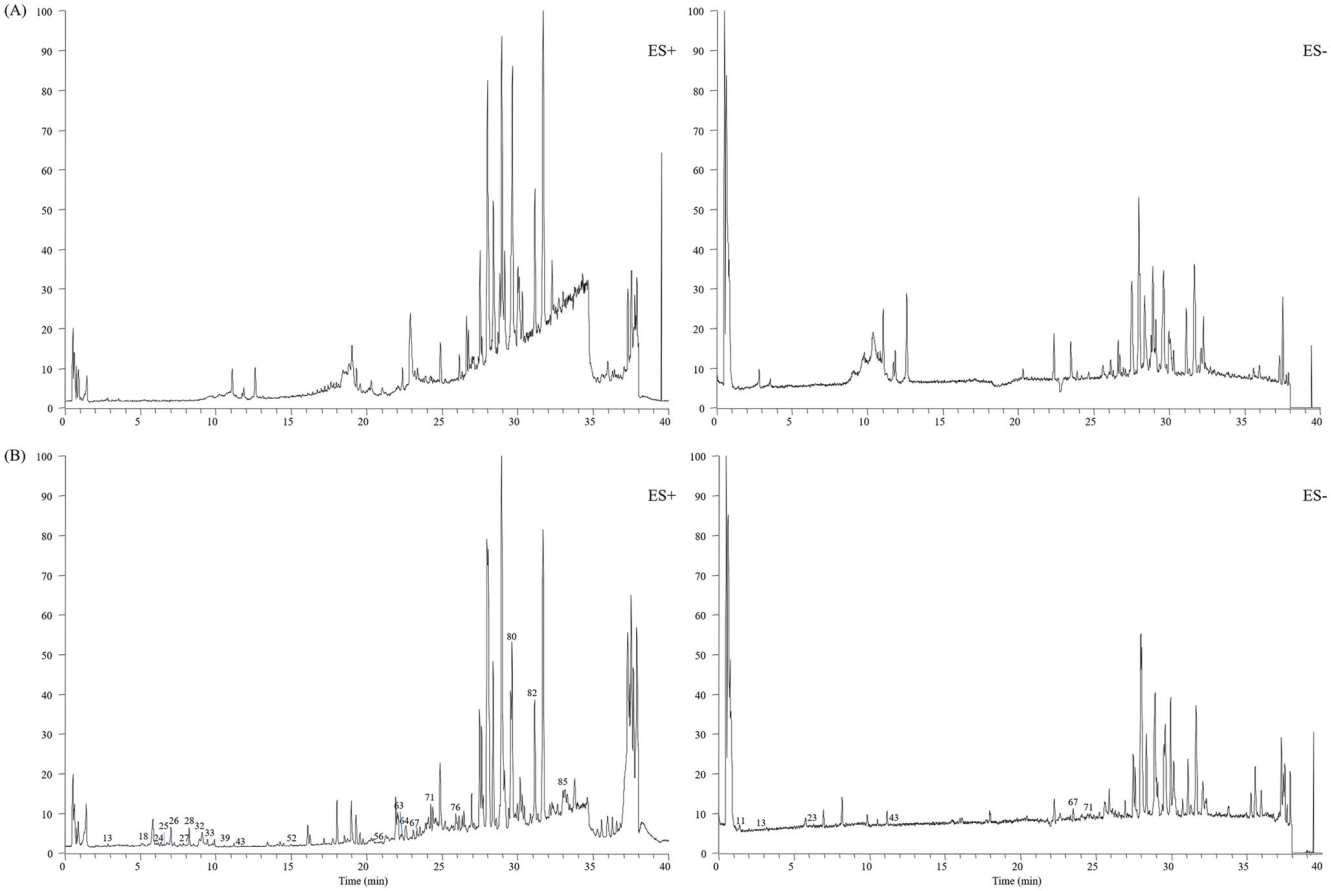
Total-ion chromatograms in the positive and negative modes of blank rat serum (A) and the serum after oral administration of ZJP (B).

In this study, the developed UPLC-HRMS method was also applied to find the constituents absorbed in rat serum after gavage of ZJP. Blank rat serum and rat serum after the oral administration of ZJP were analysed by the developed UPLC-HRMS method, and the total ion chromatograms are given in Figure 8. The chemical composition data of the ZJP including retention time, exact mass and MS/MS fragment, which were obtained from the characterisation of compounds in the ZJP *in vitro*, were used to find prototype absorbed blood components. Finally, a total of 23 prototypes in the ZJP were found in the serum after oral administration of ZJP. Among them, 13 were from CC and 10 from TR. The absorbed compounds are all marked with ^Δ^ in Table 1.

### Gastroprotective effect of ZJP on the gastric mucosa

#### Morphological findings and ulcer index

**Figure 9.**
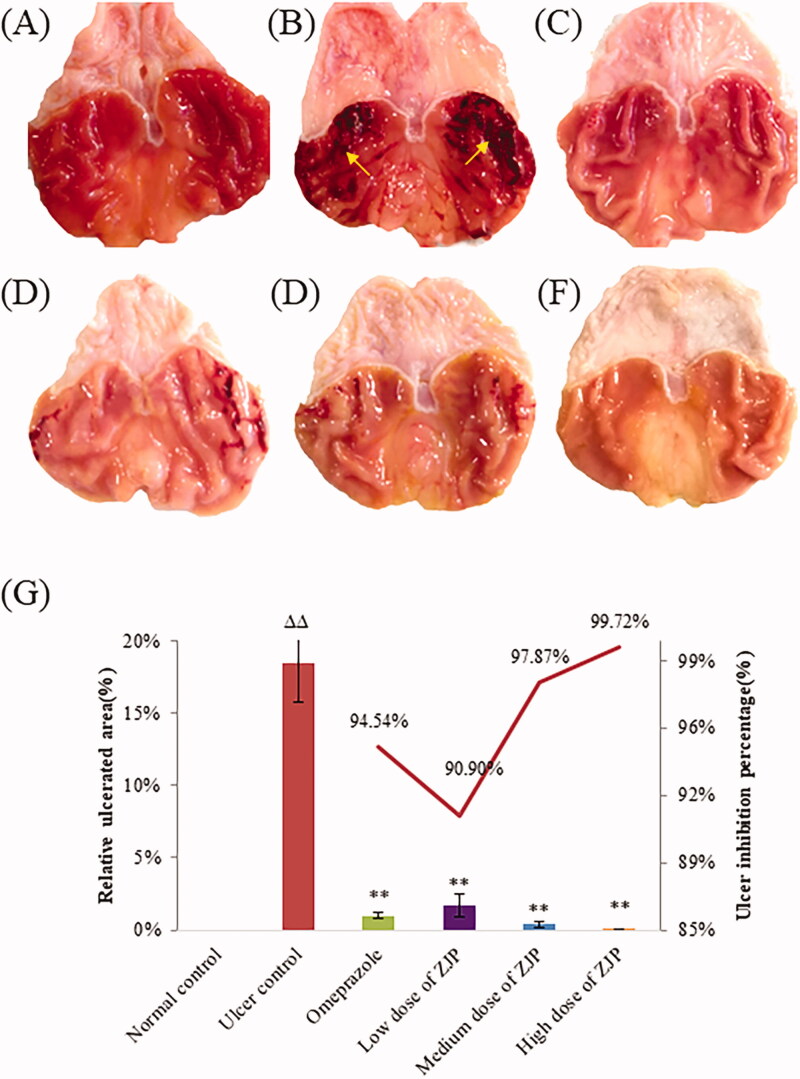
Protective effects of ZJP on the gastric mucosa evaluated by photos of the stomach: (A) normal control group, (B) ulcer control group (yellow arrows: submucosal haemorrhagic stripes), (C) omeprazole group, (D) low dose of ZJP group; (E) medium dose of ZJP group; (F) high dose of ZJP group and (G) the relative ulcerated area and ulcer inhibition percentage of various experimental groups. Δ: comparing with the normal control group, *: comparing with the ulcer control group.

Photographs of the stomachs from each group are presented in Figure 9. As shown in Figure 9A, a completely healthy pink gastric mucosa with normal thickness was found in the stomach of normal rats. The rats in the ulcer control group displayed critical tissue reactions with long dark red submucosal haemorrhagic stripes and mucosal thickening, indicating that alcohol-induced gastric ulcer was successful. Oral pre-treatment with omeprazole or different doses of ZJP weakened the gastric lesions, including their number and length. Noteworthy, haemorrhages, thickening and congestion were hardly found in the high dose of ZJP group, and the mucosa colour was pink, like normal.

To quantitatively evaluate the protective effect of ZJP, the relative ulcerated area (%) and ulcer inhibition percentage were calculated, as shown in Figure 9G. A remarkable decrease in ulcerated areas was observed after ZJP administration. The ulcer inhibition percentage was greater than 90.0% in all the ZJP groups. The results demonstrated that ZJP could protect the gastric mucosa against acute injury caused by ethanol, and was even better than omeprazole.

#### Histopathological examination

**Figure 10.**
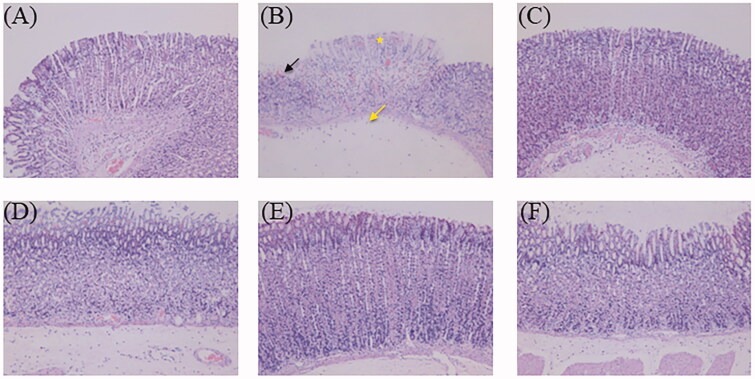
The haematoxylin and eosin-stained gastric tissue micrographs (400× magnification): (A) normal control group, (B) ulcer control group (star: necrotic area of gastric mucosa, yellow arrow: oedema of the submucosa, black arrow: haemorrhagic patches), (C) omeprazole group, (D) low dose of ZJP group; (E) medium dose of ZJP group; (F) high dose of ZJP group.

Histopathological alterations in stomach specimens by haematoxylin and eosin staining of the different groups are given in Figure 10. Histological examination of the normal control group showed undamaged villi of the gastric mucosa without exfoliation, congestion, or haemorrhages in the mucosal epithelium. The ulcer control group displayed critical injury to the gastric mucosa, such as serious cell exfoliations of the superficial epidermis, focal necrotic ulcerative areas and damaged glandular structures. In addition, neutrophil infiltration, edoema, massive haemorrhage and necrosis were noticed. Some necrotic cells were also observed in the submucosa. Moreover, the gastric juice volume of the ulcer group treated with ethanol was found to be higher than that of the normal group in this study, which was associated with acute injury to the gastric mucosa. The omeprazole-pre-treated group showed that the exosmosis of red blood cells in the gastric mucosa became negligible, with the almost intact gastric mucosa. Gastroprotective effects were observed in all the ZJP-pre-treated groups. Milder focal ulceration and smaller haemorrhagic areas of the gastric mucosa were noticed in the low dose ZJP group (1 g/kg), while the medium dose (2 g/kg) and high dose (4 g/kg) groups exhibited better gastroprotection, with normal undamaged mucosa with no haemorrhage or congestion. These results confirmed that ZJP remarkably improved ethanol-induced damage to gastric tissue, which was consistent with the morphological findings.

#### Effect of ZJP on oxidative stress in ethanol-treated rats

**Figure 11.**
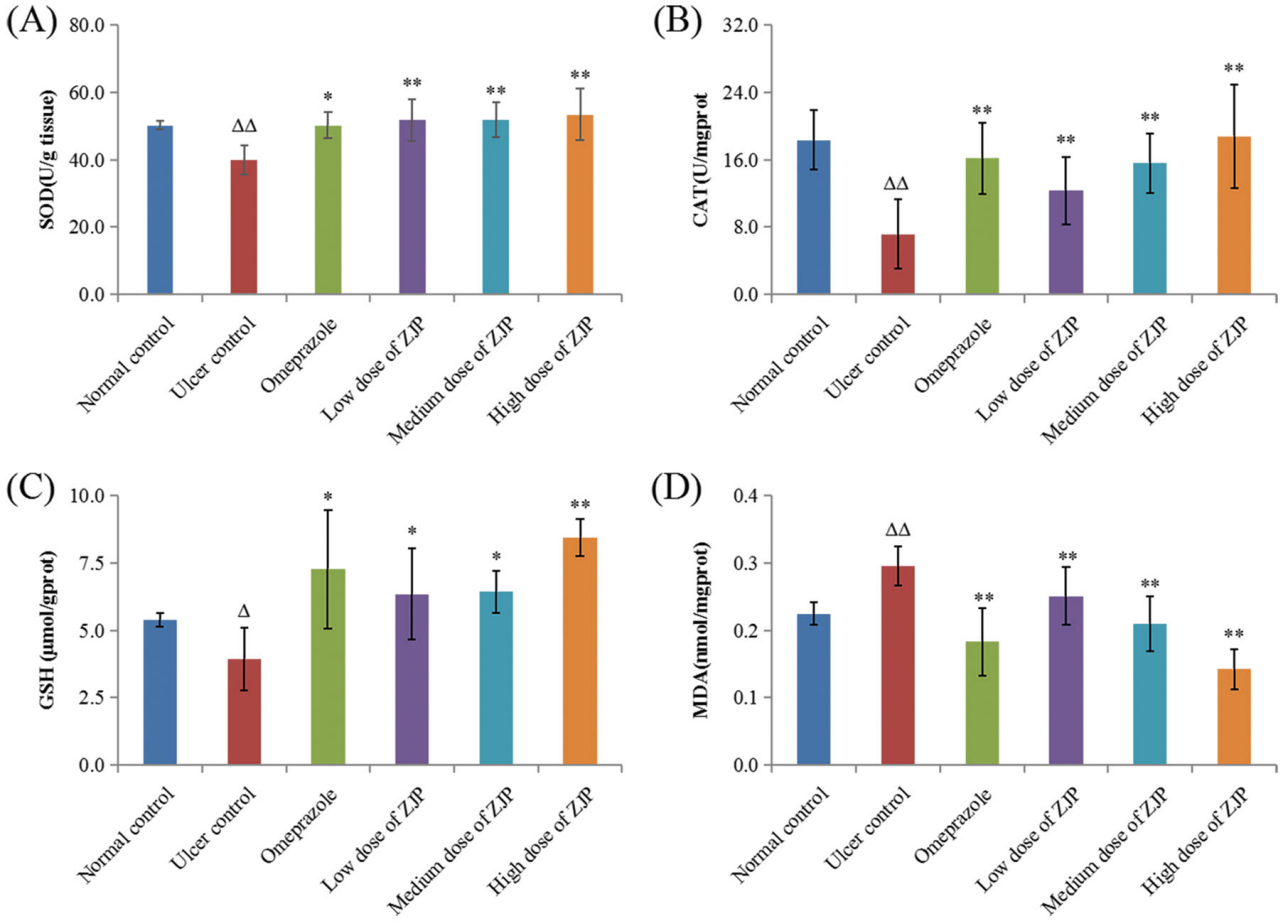
Effect of ZJP on the level of (A) SOD, (B) CAT, (C) GSH and (D) MDA in the gastric tissue of different treated groups. Δ: comparing with the normal control group, *: comparing with the ulcer control group.

The influence of ZJP on ethanol-induced oxidative stress was studied by testing SOD, CAT, GSH and MDA in different groups. In the present study, the ulcerated rats after administration of ethanol significantly reduced gastric SOD (20.5%), CAT (60.9%) and GSH (27.1%). While, the administration of ZJP (1, 2 and 4 g/kg) meaningfully improved the activity of SOD by approximately 30.0% compared with the ulcer control rats (Figure 11A). Referring to the ulcer control rats, the ZJP pre-treatment (1, 2 and 4 g/kg) evidently elevated the CAT levels by 71.9, 117.3 and 161.6%, respectively (Figure 11B). Moreover, the content of GSH in rats pre-treated with ZJP (1, 2 and 4 g/kg) substantially went up by 61.3, 63.5 and 114.4%, respectively (Figure 11C), compared to the ulcer control rats. It was noteworthy that the SOD, GSH and CAT data in the ZJP-pre-treated group (4 g/kg) were better than the respective values of the omeprazole group. The content of MDA in the ulcerated control rats was noticeably higher than that of normal rats (*p* < 0.01) (Figure 11D). While pre-treatment with ZJP (2 and 4 g/kg) significantly decreased MDA levels compared to those in ulcer rats.

#### Effect of ZJP on inflammation in ethanol-treated rats

**Figure 12.**
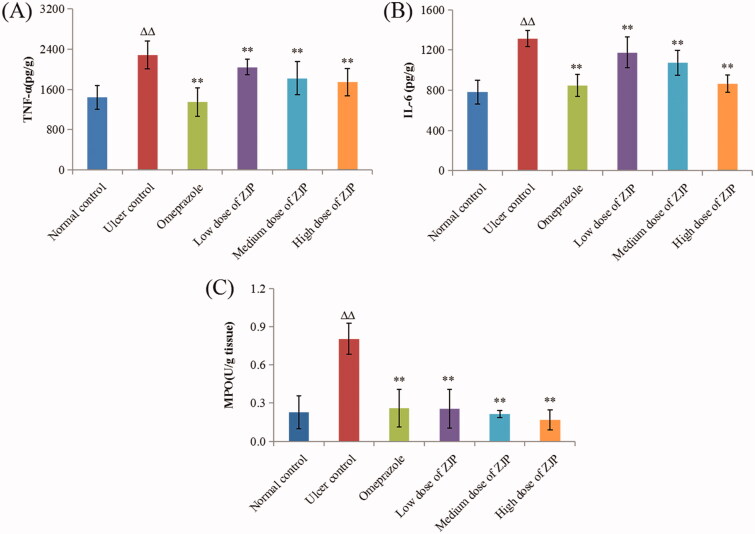
Effect of ZJP on the level of (A) TNF-α, (B) IL-6 and (C) MPO in the gastric tissue of different treated groups. Δ: comparing with the normal control group, *: comparing with the ulcer control group.

As shown in Figure 12, TNF-α and IL-6 apparently rose by 58.8% and 68.1% in the ulcer control group compared to those in the normal control group (*p* < 0.01). While the group treated with ZJP (1, 2, and 4 g/kg) displayed a noteworthy decrease in TNF-α and IL-6 content (*P* < 0.01) in a dose-dependent mode referred to ulcer model group. In addition, the content of MPO in each group was also determined to evaluate the level of gastric leukocyte aggregation induced by ethanol. The gastric MPO was discovered to surge by 250.0% in the ulcer control group referred to the normal control group, showing that there was neutrophil infiltration in the lesion. As seen in Figure 12C, pre-treatment with ZJP (1, 2, and 4 g/kg) also led to a dose-dependent reduction in the gastric MPO level compared with the ulcer model group. In particular, the degree of MPO recovered to the normal level in the high dose of ZJP group (4 g/kg).

#### Effect of ZJP on the expression of NF-κB (p65) in ethanol-treated rats

**Figure 13.**
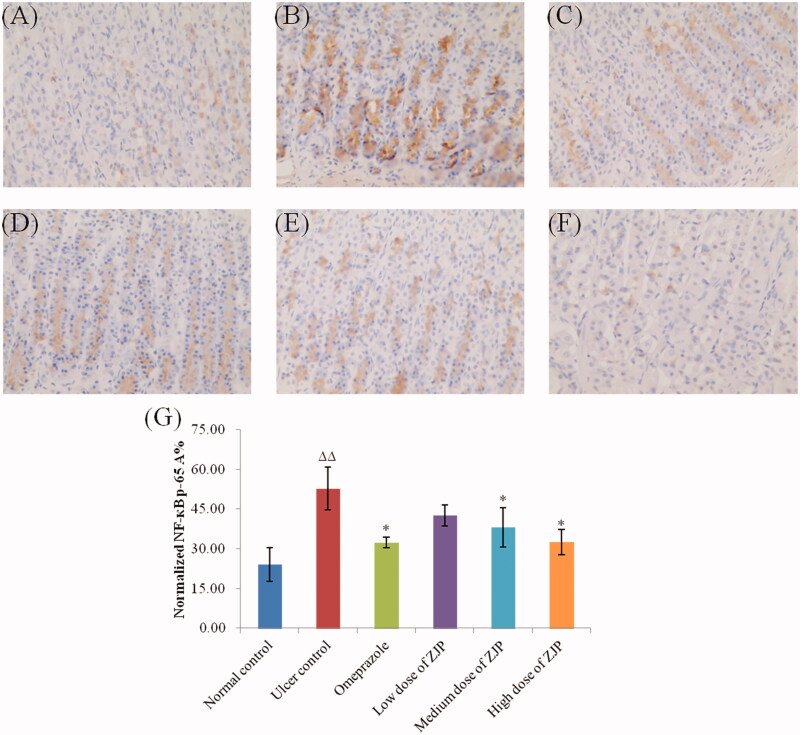
Effect of ZJP on the expression of NF-κB p65 in gastric mucosa by immunohistochemical staining (400 × magnification): (A) normal control group, (B) ulcer control group, (C) omeprazole group, (D) low dose of ZJP group; (E) medium dose of ZJP group; (F) high dose of ZJP group and (G) The normalised NF-κB p65 A%. The antigen site of NF-κB p65 appears as a brown colour (*n* = 6). Δ: comparing with the normal control group, *: comparing with the ulcer control group.

In this study, the level of NF-κB (p65) in gastric tissue was immunohistochemically detected in the different groups. As illustrated in Figure 13, an obvious surge in the expression of NF-κB (p65) was found in the ulcer control group by 118.6 referred to the normal control group. While the pre-treatment with ZJP (2, and 4 g/kg) or omeprazole resulted in a marked decrease in the expression of NF-κB (p65) by 27.7%, 38.3% and 38.6%, respectively. In addition, the inhibition of NF-κB (p65) by ZJP occurred in a dose-dependent mode.

## Discussion

The compounds of ZJP are very complex, and clarifying its chemical basis *in vitro* and *in vivo* is necessary for the clinical application and development of ZJP. Meanwhile, the mechanisms of ZJP are still unclear. So, this study aimed to reveal more potential active ingredients and mechanisms relating to the gastroprotective effect of ZJP.

Recently, various mass techniques have been used to rapidly characterise compounds in complex samples, such as HPLC-Q-TOF MS, HPLC-FTICR-MS and HPLC-Q-Orbitrap MS. The UPLC-Q-Orbitrap HRMS technique can identify trace-level compounds in sample mixtures because of its high resolution and powerful mass axis calibration (Liu et al. 2019). In this study, a UHPLC-Q-Orbitrap HRMS method was established to characterise and identify the compounds in ZJP.

In this study, a four-step structured strategy was proposed to systematically characterise and identify components in the ZJP. First, a housed library of compounds from CC and TR was built according to literature and several online databases, such as ChemSpider, mzcould, and TCM Database@Taiwan. The established library summarised the information, including the compound name, structure, chemical formula and molecular weight. Additionally, representative components of different types of compounds were analysed by HRMS to obtain mass spectral fragmentation pathways and diagnostic ions. Usually, compounds with identical carbon skeletons can produce characteristic homologous fragment ions in mass spectrometry. Subsequently, the target compounds of the ZJP were quickly identified by diagnostic ions, and the structures of the compounds were further identified by precise molecular weight and fragmentation pathways. At the same time, other high-response chromatographic peaks in ZJP samples were identified through precise molecular weight and fragmentation pathways based on our established compound library. Finally, the fragmentation mechanism of the compounds was elucidated. Based on the proposed four-step strategy, 90 compounds were identified or tentatively characterised in the ZJP. Thus, the *in vitro* chemical basis of ZJP was clarified, involving alkaloids, flavonoids, phenolic acids, and limonoids. Furthermore, alkaloids included protoberberine alkaloids, aporphine alkaloids, indole alkaloids, quinolone alkaloids, and other alkaloids. Additionally, the proposed strategy could also be applied to identify components of other plants or traditional Chinese medicine.

Oral administration is the basal mode of action in traditional Chinese medicine. Bioactive ingredients would be absorbed into the blood and transferred to the target. Thus, in terms of serum pharmacochemistry, compounds that could be absorbed into circulation might be the active ingredients for complex herbal formulations (He et al. 2015; Zhang et al. 2016). So, the constituents absorbed in rat serum after gavage of ZJP were furtherly clarified using the developed UPLC-HRMS method. 23 prototypes in the ZJP were found in the serum after oral administration of ZJP, which might be the pharmacodynamic material basis *in vivo*, including jatrorrhizine, berberine, dehydroevodiamine, and evodiamine and so on. Recent references have proved that these alkaloids from CC and TR have various activities including antioxidant, anti-inflammatory, and anticancer activities (Tian et al. 2019; Li et al. 2020).

After clarifying the chemical basis of ZJP *in vitro* and *in vivo*, the gastroprotective effects of ZJP were evaluated in the rat model of ethanol-induced gastric injury. The recommended human dose of ZJP is 6–12 g/day according to the Chinese Pharmacopoeia (2020). And the administered dose of ZJP in the rat study was 0.6–5.04 g/kg in the early references (Wang et al. 2020; Tong et al. 2021; Wei et al. 2021). So, 1.0, 2.0 and 4.0 g/kg was determined as a low, medium and high dose of ZJP in this study, respectively. In studies evaluating the antiulcer efficacy of potential medicine, ethanol, NSAIDs or pylorus ligation have been usually used to induce gastric ulcers in the animal model (Meng et al. 2019). Ethanol can easily infiltrate into the gastric mucosa to damage the defensive mucous, producing acute injuries (Ahmed et al. 2022). Oral administration of absolute ethanol in experimental animals could reproducibly induce gastric ulcers, which is similar to alcohol injury in humans (Hobani et al. 2022). So, in this study, the anti-gastric ulcer effect of ZJP was evaluated in a rat model induced by oral administration of a single dose of 5 mL/kg absolute ethanol. The macroscopical and histopathological experiments both revealed that ethanol administration led to severe gastric mucosal damage, such as submucosal haemorrhagic stripes, focal necrotic ulcerative areas and damaged glandular structures. These findings are similar to earlier studies (Fahmy et al. 2020; Jafari et al. 2022). In contrast, pre-treatment with ZJP lighten these damages, and the ulcer inhibition percentage was greater than 90.0% in all the ZJP groups. These results proved ZJP could significantly lighten gastric ulcer-induced ethanol in rats.

Oxidative stress is a primary factor contributing to mucosal lesion formation (Meng et al. 2019). Ethanol can cause gastric tissue damage by inducing ROS production (Mousa et al. 2019). Afterward, the protective antioxidant defense mechanisms would be depleted and lipid peroxidation would increase. In this process, the activity of antioxidative enzymes including SOD and CAT and the concentration of GSH would decrease, while the lipid peroxidation products (MDA) would increase. The result would cause damage to mitochondria and lysosomes, and stimulate the development of ulcers. Compounds with antioxidative activity have been proved to prevent gastric mucosa from ulcers (Tahir et al. 2022). The antioxidative activity of ZJP was evaluated in this study. The result revealed that administration of ZJP could obviously improve the activities of antioxidant enzymes (SOD and CAT), restore the depleted GSH content, and decrease the MDA level, thereby protecting the gastric mucosa against ethanol-induced ulcers. The antioxidant effect of ZJP at the high dose was more significant than that of omeprazole. Therefore, the gastroprotective activity of ZJP may be partly associated with its repressive effect on oxidative stress and lipid peroxidation.

Oxidative stress has been reported to induce inflammatory responses, while inflammatory responses were also related to the pathogenesis of ethanol-induced ulceration (Al-Sayed et al. 2020). Ethanol consumption can stimulate a surge in IL-6 and TNF-α (Lv et al. 2019). TNF-α secreted by migrating macrophages in inflammation irritates the infiltration of neutrophils in the gastric inflammation area, inhibits microcirculation in the gastric ulcer mucosa, and postpones the healing of gastric ulcers. IL-6 can induce the aggregation of neutrophils and ultimately lead to an increase in inflammatory mediators. The level of IL-6 and TNF-α is correlated with the extent of inflammation in gastric mucosal (Wang et al. 2015). Additionally, MPO, secreted mainly by neutrophils, regulates the inflammatory response. Compounds that reduce the level of MPO might have anti-inflammatory bioactivity (Lv et al. 2019). In this study, pre-treatment with ZJP (1, 2, and 4 g/kg) led to a dose-dependent decrease in the contents of TNF-α, IL-6 and MPO compared with the ulcer model group. The results demonstrated that ZJP suppressed the inflammatory response to alleviate ulcers in gastric tissue by decreasing the content of TNF-α and IL-6 and restraining neutrophil infiltration of the ulcer gastric tissue. These results testified to the anti-ulcer effect of ZJP via an anti-inflammatory mechanism. This is consistent with the histopathological experiments, which showed that the inflammatory response was reduced after ZJP pre-treatment.

NF-κB is a “fast-acting” primary transcription factor, which is involved in the response of cells to cytokines, oxidative stress or other stimuli (Lawrence 2009). Moreover, NF-κB is a typical pro-inflammatory signalling pathway, which can be activated by interleukins, TNF-α or other pro-inflammatory cytokines (Al-Sayed et al. 2020). The expression of NF-κB (p65) was found to decrease obviously after administration of ZJP. The results showed that ZJP downregulated the expression of pro-inflammatory cytokines/chemokines by inhibiting the activation of NF-κB induced by TNF-α or interleukins, thereby exerting an anti-inflammatory effect in the gastric tissue. These results were consistent with those of the pro-inflammatory cytokine experiments.

These results in ethanol-treated rats proved that ZJP could mitigate gastric ulcers induced by ethanol via antioxidant and anti-inflammatory mechanisms. And these activities of ZJP might be related to the identified compounds. UPLC-Q-Orbitrap HRMS experiments showed that ZJP was composed of various alkaloids, flavonoids and limonoids. Alkaloids from CC, such as berberine, jatrorrhizine and magnoflorine, could inhibit the classical inflammatory pathways by mediating NF-κB and AP-1 (Xu, Kuang, et al. 2020; Xu, Zhang, et al. 2020). Early studies have proved that dehydroevodiamine, evodiamine and rutaecarpine, isolated from the ethanol extract of TR, could diminish the production of ROS and NADPH oxidase activity and reduce the activation of NF-κB (Ko et al. 2007; Tian et al. 2019). Furthermore, the literature indicated that limonin characterised by ZJP exhibited protective activities against long-term alcohol injury due to antioxidant and anti-inflammatory mechanisms (Valansa et al. 2020). Based on these results in this study and the literature on various compounds in ZJP, it can be concluded that the gastroprotective activities of ZJP were attributed to the cooperation of its multiple compounds identified by UPLC-Q-Orbitrap HRMS in this study.

## Conclusion

This study established an approach involving UPLC-Q-Orbitrap HRMS and serum pharmcochemistry to screen the multiple chemical constituents of ZJP. Diagnostic ions and accurate mass measurements were used to identify the target compounds. A total of 90 components, including 58 alkaloids, 11 flavonoids, 7 phenolic acids, 3 amino acids, 8 limonoids and 3 other type components, were identified or tentatively characterised in the ZJP. Among them, 23 prototypes in the ZJP were detected in rat serum after oral administration, which was the potential active ingredients. Furthermore, the results of the pharmacodynamic study demonstrated that ZJP could mitigate gastric ulcers induced by ethanol via antioxidant and anti-inflammatory activities. The therapeutic effects of ZJP on gastric ulcers might benefit from the synergistic actions of multiple ingredients. These results could provide an experimental basis to support the clinical application of ZJP in gastric ulcer therapy.
